# Failure Analysis of Cracked P110 Repaired Tubing Used for Gas Transmission

**DOI:** 10.3390/ma16227151

**Published:** 2023-11-14

**Authors:** Shuxin Zhang, Faqin Xie, Xiangqing Wu, Xi Yan, Jinheng Luo, Xiaoliang Ma, Gege Su

**Affiliations:** 1 School of Civil Aviation, Northwestern Polytechnical University, Xi’an 710072, China; 2 Tubular Goods Research Institute, China National Petroleum Corporation & State Key Laboratory for Performance and Structure Safety of Petroleum Tubular Goods and Equipment Materials, Xi’an 710077, China; 3 Shaanxi Society for Environmental Sciences, Xi’an 710000, China; 4 Tarim Oilfield Company, PetroChina, Co., Ltd., Korla 841000, China

**Keywords:** repaired tubing, P110, crack, thermal simulation technique, weldability

## Abstract

With green and low-carbon developments in oil fields, an increasing amount of repaired oil tubing is being used as oil and gas transmission pipelines in China. However, due to differences in manufacturing standards between oil tubing and transmission pipelines, there are inevitably some issues during their use. This paper investigates a case of cracking failure in repaired oil tubing used as a gathering and transportation pipeline. The failure occurred after eight months of operation and was characterized by a circumferential crack at the male thread end of the tubing joint. To determine the root cause of the failure, a series of experiments were conducted on the oil tubing. The experiments included visual inspection, chemical composition analysis, mechanical properties testing, hardness testing, metallographic examination, and microstructure analysis. The results revealed that the thread of the cracked tubing was not tightened to the specified position; the connection between the tubing and the coupling was welded in a circumferential direction; and cracks occurred in the heat-affected zone of the weld. Chemical composition, tensile performance, and the Charpy impact of the tubing meet the requirements of API 5CT for P110 material, and no abnormalities were found in the metallographic structure. The microstructure at the weld toe of the fracture is martensite, and the hardness is 476 HV10. Based on the thermal simulation verification test, when the material of the tubing cools from 1200 °C, which is located in the coarse HAZ temperature zone, the base metal transforms into martensite with a little granular bainite, exhibiting its highest hardness value at 371 HV10, which is higher than the allowable hardness for carbon steel and indicates the material has poor weldability. The reasons for the cracking and failure of the tubing are that the P110 repaired tubing has a high carbon equivalent and poor weldability. During the welding process, martensitic structure was formed at the weld toe, and cold cracks appeared in the heat-affected zone, resulting in failure. To avoid the reoccurrence of such failure, recommendations are proposed.

## 1. Introduction

With the development of oilfield exploitation, the water content of oil wells continues to increase, even reaching 90% [1,2]. Oil well exploitation methods have also changed from traditional water injection [3] or gas injection to CO_2_ injection [4,5,6] and polymer injection for oil exploitation. As a result, the exploitation environment has become increasingly harsh; the working conditions have become increasingly complex; the difficulty of crude oil extraction has increased year by year; and the failure of tubing has become increasingly serious [7,8]. During inspections, some of the oil tubing is replaced. The replaced tubing may not be suitable for the current working conditions but should not be discarded, as it is an obvious waste of resources. In order to revitalize waste materials, reduce waste of resources, and realize green and low-carbon production in China, waste oil tubing (named as repaired tubing), especially largely used P110 and C110 tubing, is mainly used by oil fields as gathering and transportation pipelines for water injection and gas injection or temporary transportation, which reduces production costs and improves economic benefits.

However, during the servicing process, oil tubing is subject to corrosive media and complex loads and is prone to eccentric wear [9], uniform corrosion [10], pitting corrosion [11], stress corrosion cracking [12], and other failures. When used as a gathering and transportation pipeline, systematic defect detection must be carried out to ensure the safety of transportation. In addition, oil tubing is usually connected by thread, while the transmission pipeline is usually connected by welding, so the pipeline delivery pressure should not be too high. In addition, when the thread connection fails, how to plug and repair it is the main problem faced.

In this study, a cracking failure case of repaired oil tubing used as a gathering and transportation pipeline was studied. Such failure cases are rarely reported. Therefore, these research conclusions have great reference value for the application of repaired oil tubing as gathering and transportation pipelines.

**Figure 1:**
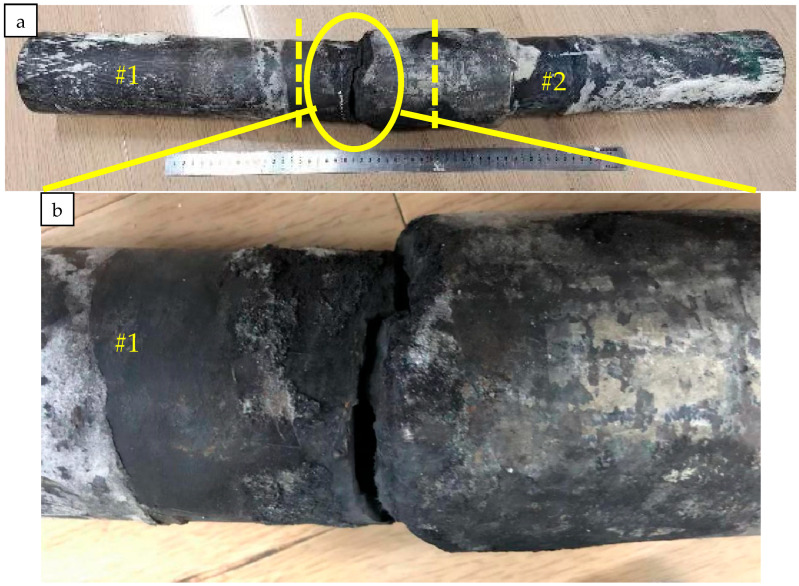
Macroscopic morphology of failed pipe section. (**a**) The whole pipe (**b**) The cracked section.

The studied repaired oil tubing used as a gathering and transportation pipeline leaked after being put into operation for eight months. The oil tubing specification is Φ88.9 mm × 6.45 mm. The material is P110 or C110 according to API 5CT, and the transmission medium is natural gas; the gas transmission volume is about 100,000 cubic meters per day; the service temperature is 15 °C; the operating pressure is 5 MPa. According to the transmission medium composition test results, the medium contains 0.7669% H_2_S and 2.387% CO_2_. The leakage region of the oil tubing is the joint, and a circumferential crack occurred at the male thread end, as shown in Figure 1. To find the root cause of the failure, a series of experiments were carried out on the oil tubing.

## 2. Experiment and Method

The total length of the cracked repaired oil tubing is about 100 cm, including two sections (marked as #1 and #2 oil tubing, respectively) and coupling. Test samples were taken from the #1 and #2 repaired oil pipes and coupling, and the chemical composition of the samples was analyzed by an ARL4460 direct-reading spectrometer (Thermo ARL, Waltham, MA, USA) according to the ASTM A751-20 standard. A tensile test of the #1 and #2 tubing was carried out on longitudinal tensile samples, according to the ASTM A370-20 standard with a UTM5305 material testing machine (SUNS, Shenzhen, China). The size of the longitudinal Charpy impact test specimen is 5 mm × 10 mm × 55 mm with a V-shaped notch, and the impact test was carried out by a PIT302D impact testing machine (WANCE, Shenzhen, China) according to the ASTM A370-20 standard at room temperature. The Rockwell hardness test was undertaken on the #1 and #2 tubing rings according to ASTM E18-20 with an RB2002T Rockwell hardness tester (Wilson, IL, USA). The micro-Vickers hardness test was undertaken on the failed area of the tubing to check whether there is a detrimental phase with the DuraScan 70G5 hardness tester (Emcotest, Kuchl, Austria)according to the standard ASTM E384-17.

The metallographic samples were machined from the #1 and #2 repaired tubing, the coupling, and the failed region, and their microstructure was inspected with an MEF4M metallographic microscope (Leica, Wein, Austria) according to ASTM E45-18a, ASTM E1268-19. The morphology of the cross section of the cracked region was observed under a scanning electron microscope of Tescan Vega (Brno, Czech Republic). Energy dispersive spectroscopy was adopted to check the element content of the corrosion products on the fracture surface.

The weld simulation was conducted on the base metal of the failed #1 repaired tubing on a Gleeble 3500 thermal simulation testing machine (Dynamic Systems Inc., New York, NY, USA) to study the different heat-affected zone (HAZ)s’ microstructure and hardness properties. The dimensions of the sample are Φ4 × 100 mm; the base metal was heated to 850 °C, 950 °C, 1050 °C, 1100 °C, and 1200 °C, respectively, kept for 3 s, then air-cooled to room temperature. And the load was recorded, and the microstructure was observed.

## 3. Experiment Results

### 3.1. Visual Inspection

**Figure 2:**
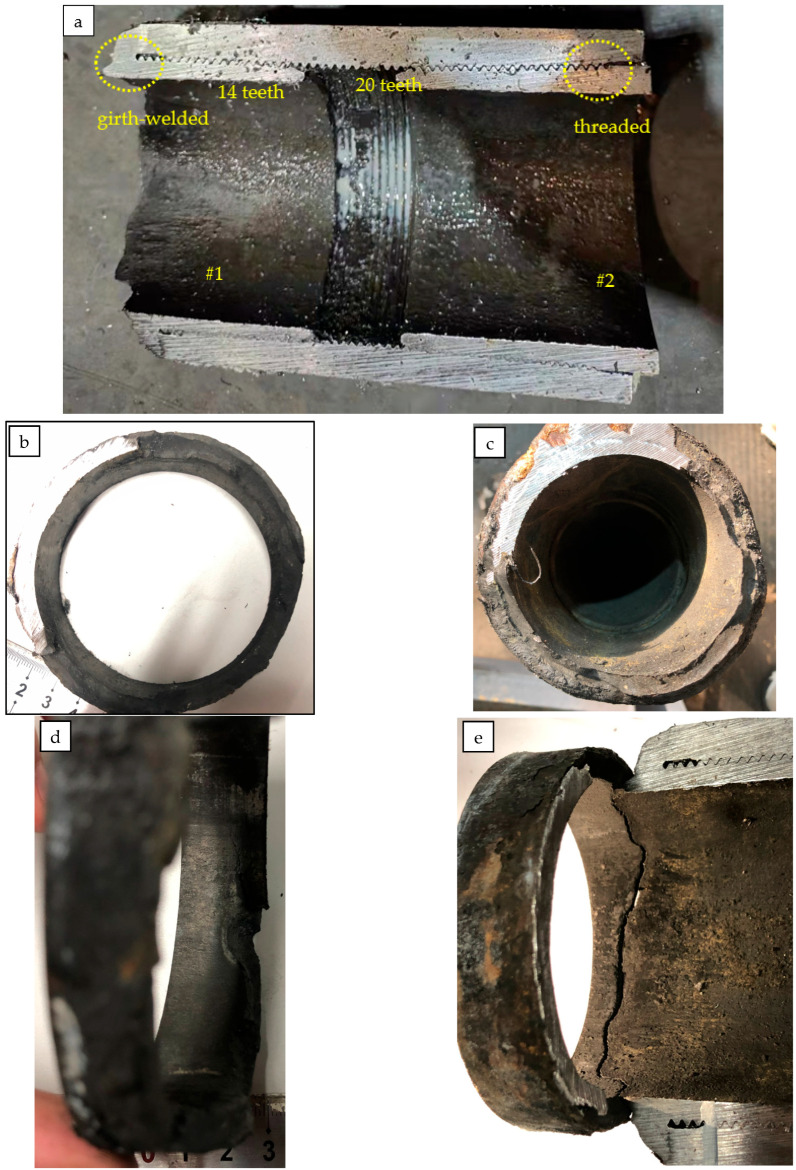
Macroscopic morphology of fracture.

The failed repaired tubing at the coupling was cut in half, as shown in Figure 2; the #1 repaired tubing and the coupling were girth seal-welded at the connection region, while the #2 repaired tubing and the coupling were threaded.

The circumferential crack is located near the girth weld of the #1 tubing; the inner surface of the coupling joint is covered with black oil; and there is no obvious corrosion on the coupling and the #1 and #2 tubing. From the cross-section view, there are 20 teeth in total for the #2 male thread tubing, and about 14 teeth for the #1 male thread tubing. Therefore, it can be inferred that the male thread end of the #1 tubing has not been threaded to the specified position. The surface of the fracture is covered with black matter (Figure 2b,c). Observed from the side view, there is no obvious plastic deformation at the crack of the #1 tubing.

### 3.2. Chemical Composition

Chemical composition analysis results are shown in Table 1. Since the specification of the repaired oil pipe is not clear, the chemical composition analysis results are used to determine its material type. Based on the test result, it is inferred that the #1 oil tubing is made of P110 material, and the #2 oil tubing is made of C110 material.

### 3.3. Mechanical Properties Test

**Figure 3:**
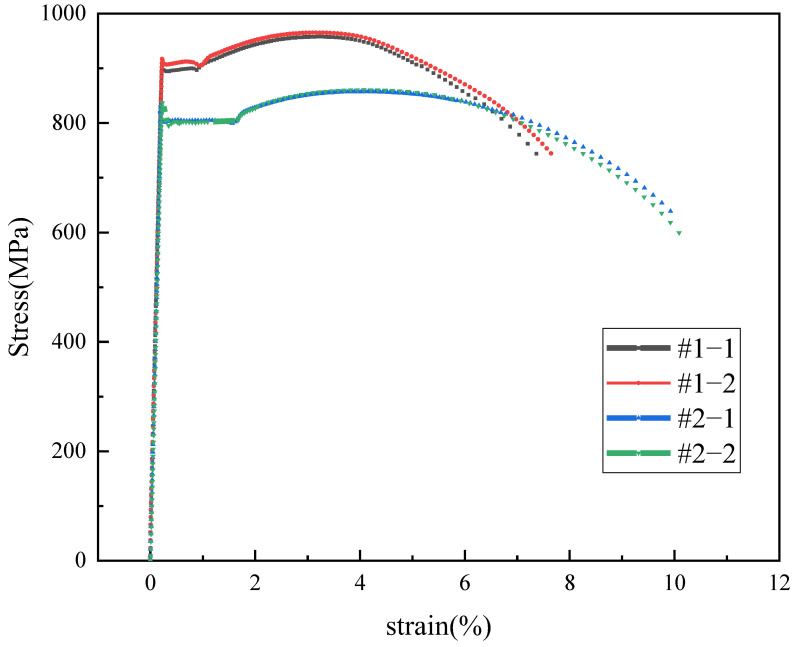
The strain–stress curve of the tensile test.

The results of the tensile test are shown in Table 2, and the strain–stress curves are shown in Figure 3. The tensile strength and yield strength of the #1 repaired tubing meet the requirements of the standard API 5CT for P110, and the tensile strength and yield strength of the #2 repaired tubing meet the requirements of the standard API 5CT for C110.

Due to the brittleness cracking of the pipes, Charpy impact testing was conducted on the pipe body to assess its fracture toughness. The results of the Charpy impact test are shown in Table 3. The longitudinal impact absorption energy of the #1 and #2 repaired tubing meets the standard API 5CT requirements.

**Figure 4:**
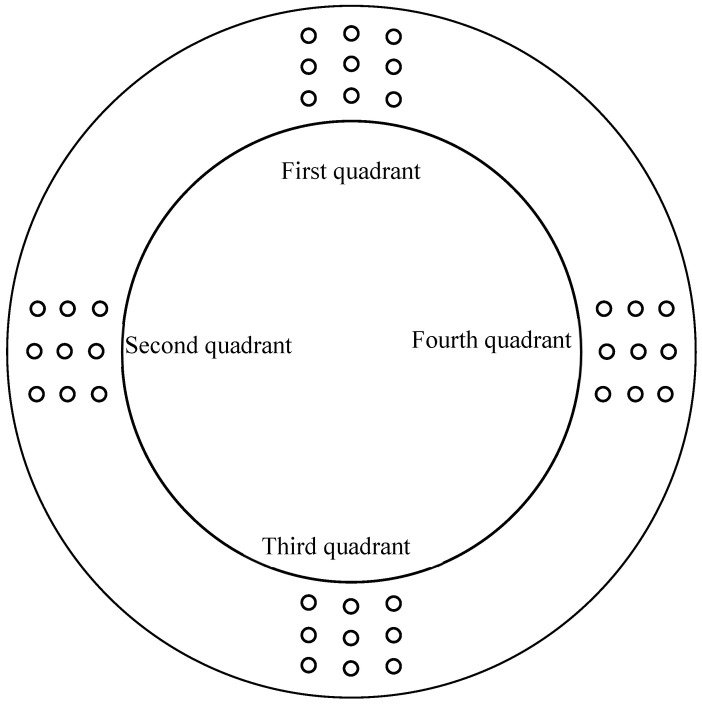
The schematic diagram of hardness test on the pipe.

The schematic diagram of the Rockwell hardness test on the pipe shown in Figure 4, and the results of the hardness test are shown in Table 4. The standard API 5CT has no requirement for the hardness of the P110 material of the #1 repaired oil pipe, and the hardness of the #2 repaired oil pipe meets the upper limit of 30HRC required by the standard for the material of C110.

**Figure 5:**
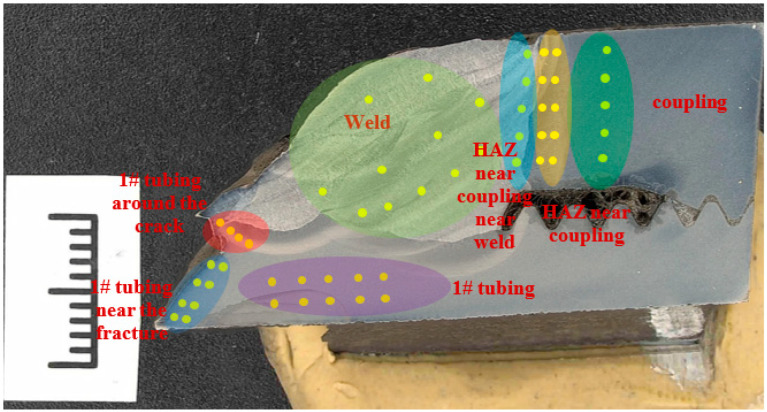
Schematic diagram of hardness test at cracked region.

The microhardness of the longitudinal section of the crack region was tested with a DuraScan 70G5 micro-Vickers hardness tester (Emcotest, Kuchl, Austria) according to the ASTM E384-17 standard (Figure 5). The results are shown in Table 5. The hardness of the coupling is between 279 and 334 HV10, and the local hardness of the heat-affected zone of the coupling increases, reaching a maximum of 391 HV10. The hardness of the welding zone is lower than that of the coupling and the tubing, which is about 220 HV10. The hardness of the #1 tubing is about 320 HV10. At the crack of the welding toe of the #1 tubing, the hardness value reaches 476 HV10.

### 3.4. Microstructure

**Figure 6:**
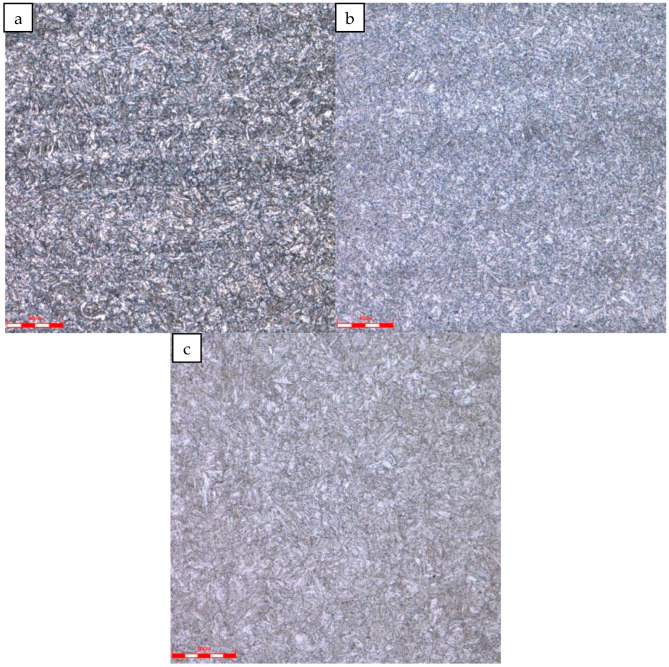
Microstructure of tubing and coupling: (**a**) #1 repaired tubing, (**b**) #2 repaired tubing, (**c**) coupling.

The metallographic structure of the sample was observed under a metallographic microscope. The microstructure of the repaired tubing and coupling was tempered sorbite, and the grain size was 9.5. The microstructure morphology is shown in Figure 6.

**Figure 7:**
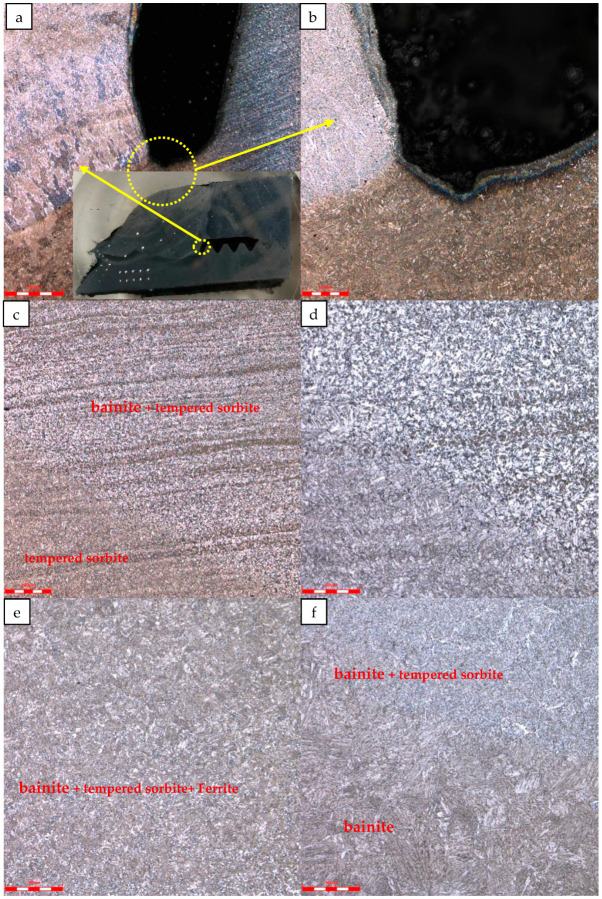
Metallographic structure of weld and fracture region: (**a**) Low magnification of welding microstructure of thread root of fracture; (**b**) The high magnification of the thread root welding microstructure of the fracture; (**c**) The low magnification of the heat-affected zone of the tubing; (**d**) High magnification of the heat-affected zone of the tubing; (**e**) Microstructure of the heat-affected zone of the tubing; (**f**) Weld and fusion zone structure of the heat-affected zone of the tubing.

Figure 7 shows the microstructure of the weld and fracture region. The tubing cracked at the heat-affected zone. Flux microstructure in the thread root welding zone of the fracture section is columnar grain, and the thread microstructure is tempered sorbite. The structure of the heat-affected zone of the tubing is tempered sorbite and bainite with a small amount of ferrite. The fusion zone is bainite. No abnormal microstructures were found.

**Figure 8:**
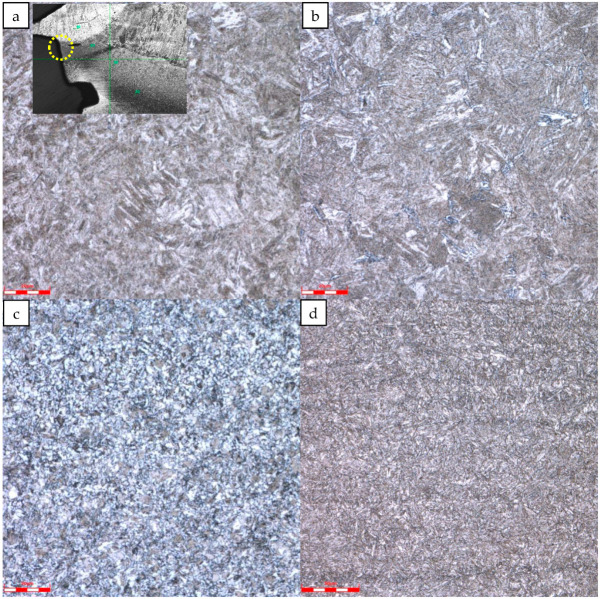
Metallographic structure near the fracture: (**a**) Microstructure at the weld toe; (**b**) Microstructure of heat-affected zone near the weld; (**c**) Microstructure of heat-affected zone near tubing; (**d**) The microstructure of the tubing base metal.

The microstructure near the fracture and the base metal was further analyzed, as shown in Figure 8. At the weld toe of the fracture, the position with the highest micro-Vickers hardness of 476 HV10, the microstructure is martensite; the microstructure in the heat-affected zone of the tubing near the weld is granular bainite; and the microstructure in the heat-affected zone near the tubing is polygonal ferrite with a Martensite/Austenite island; the tubing base metal structure is tempered sorbite.

### 3.5. Scanning Electron Microscope Test

**Figure 9:**
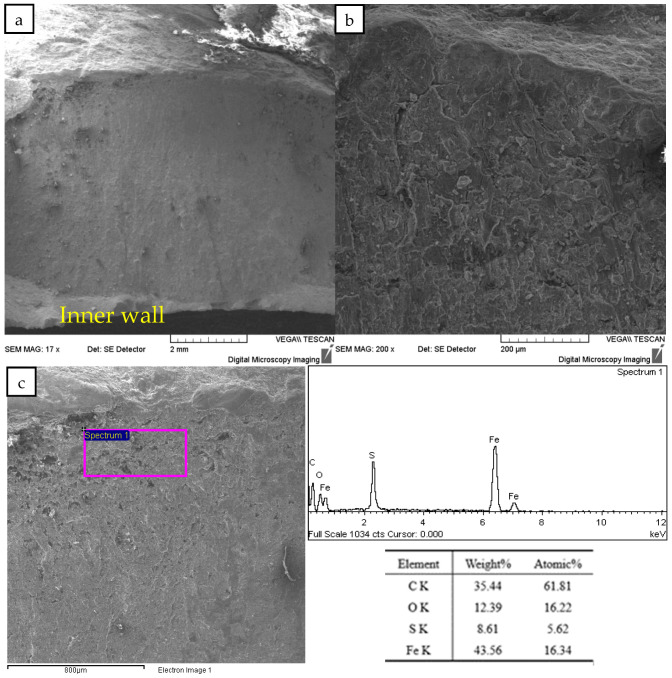
Cross-section morphology and energy disperse spectrum results: (**a**) Low magnification of crack source region; (**b**) High magnification of crack source region; (**c**) Schematic diagram and results of EDS.

The cross-sectional morphology of the fracture was observed under a scanning electron microscope, as shown in Figure 9. The fracture is flat with radial patterns on the cross section converging on the outer wall. There is a shear lip on the inner wall, which is the characteristic of the arrest area of the fracture, so it is inferred that the outer wall is the origin of the crack. Observed under high magnification, the fracture surface is covered with corrosion products, and no dimple ductile fracture characteristics are found. The surface corrosion product was characterized by an energy disperse spectrum, and the corrosion product contained C, O, S, and Fe elements, which was consistent with the H_2_S and CO_2_ contained in the medium, indicating that the crack was corroded by H_2_S and CO_2_.

## 4. Comprehensive Analysis

The macroscopic analysis of the failed tubing shows that there are welding indications at the connection between the #1 repaired tubing and the coupling; the failure crack is located at the weld, and the fracture has no obvious plastic deformation. The mechanical and chemical composition test results of the failed tubing show that the #1 repaired oil pipe is made of P110, and the #2 repaired tubing is made of C110. The chemical composition and tensile properties of the two tubes meet the standard requirements, and there is no abnormality in the Charpy impact performance.

According to the fracture analysis, the crack of the #1 repaired tubing originated from the outer wall, so the failure caused by internal corrosion of H_2_S and CO_2_ can be ruled out. From the cross-sectional metallographic view, the crack propagates along the heat-affected zone, so it is inferred that the root cause of the cracking is related to welding.

Typically, repair tubing is threaded and not welded. Generally, repaired tubing is used for water and oil transportation or associated gas transmission pipelines with a pressure not greater than 1.5MPa. Since the repaired oil pipe is manually threaded, when it is used as a pipeline, the sealing is difficult to guarantee. When the gas transmission pressure is too high, there may be leakage.

According to the information provided by operational personnel, this pipeline leaked during the pressure test and was resealed by welding. Usually, the tendency of the material to form cracks during welding and the performance of the welded joint are used as the main indicators for evaluating the welding performance of the material. The quality of weldability is related to the chemical composition of the material and the welding process used. As for steel, the carbon content has the greatest influence on weldability, so the carbon content in steel is often used as the main indicator for judging the weldability of steel materials. The higher the carbon content, the worse the weldability.

### 4.1. Weldability Analysis

Generally, there are several methods for evaluating the weldability of materials, including the implant test, the Y-slit type cracking test, the thermal simulation method, the carbon equivalent evaluation method, and the maximum hardness evaluation method. We employed the carbon equivalent evaluation method and the thermal simulation method to assess the weldability of the tube.

The International Institute of Welding (IIW) uses carbon equivalent value (CEV) [13,14] to evaluate weldability. When CE ≤ 0.45%, the weldability is good; when CEV = 0.45~0.5%, the weldability is slightly poor, and proper preheating is required before welding; when CE ≥ 0.5%, the weldability is poor, and it is a difficult-to-weld material, and a higher preheating temperature and strict welding process are required.

(1) $$\mathrm{CEV}=W\left(C\right)+\frac{W\left(\mathrm{Mn}\right)}{6}+\frac{W\left(\mathrm{Cr}\right)+W\left(\mathrm{Mo}\right)+W\left(V\right)}{5}+\frac{W\left(\mathrm{Ni}\right)+W\left(\mathrm{Cu}\right)}{15}$$

**Figure 10:**
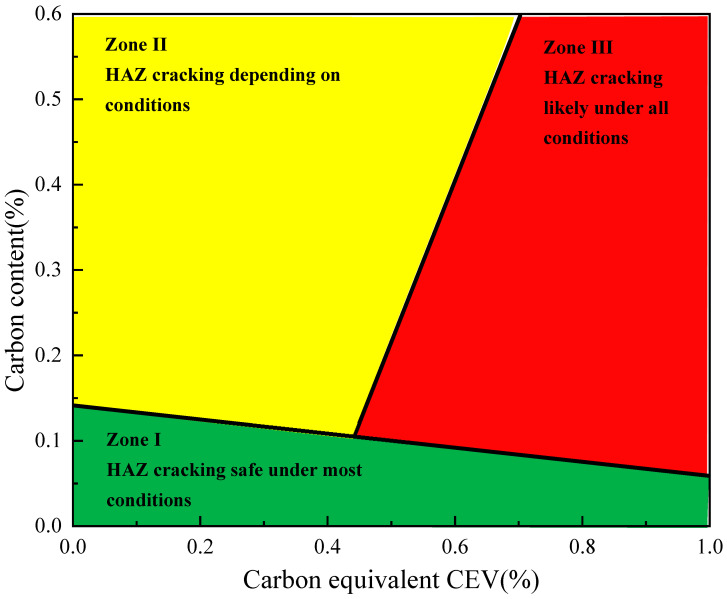
Cracking tendency—Graville diagram of welding. Black square indicated cracking tendency of #1 repaired tubing.

According to Equation (1), the carbon equivalent value of the #1 repaired tubing is calculated as 0.5369%, which indicates that the #1 repaired tubing has poor weldability and is a difficult-to-weld material. According to the cracking tendency Graville diagram [15,16] of the welding heat-affected zone as shown in Figure 10, the #1 repaired tubing material is located in the cracking zone II of the heat-affected zone, close to zone III, and is a possible cracking material.

### 4.2. Thermal Simulation Test

Experimental methods for evaluating the susceptibility of weld joints to cold cracking include the Y-slit type cracking test, the tensile restraint cracking test, the restrained butt joint cracking test, the window type restraint cracking test, controlled thermal severity, and an implant test. These methods are based on different restraint conditions and involve observing post-weld cracking. Additionally, thermal simulation is used to study the tendency for weld seam cracking [17]. Due to the complexity of the constrained conditions in samples, which are pipes in this case, the thermal simulation method is employed to investigate the microstructure of different regions in the weld and the evolution of maximum hardness. The assessment of cold cracking susceptibility in welding is based on the maximum hardness test evaluation method [18,19].

**Figure 11:**
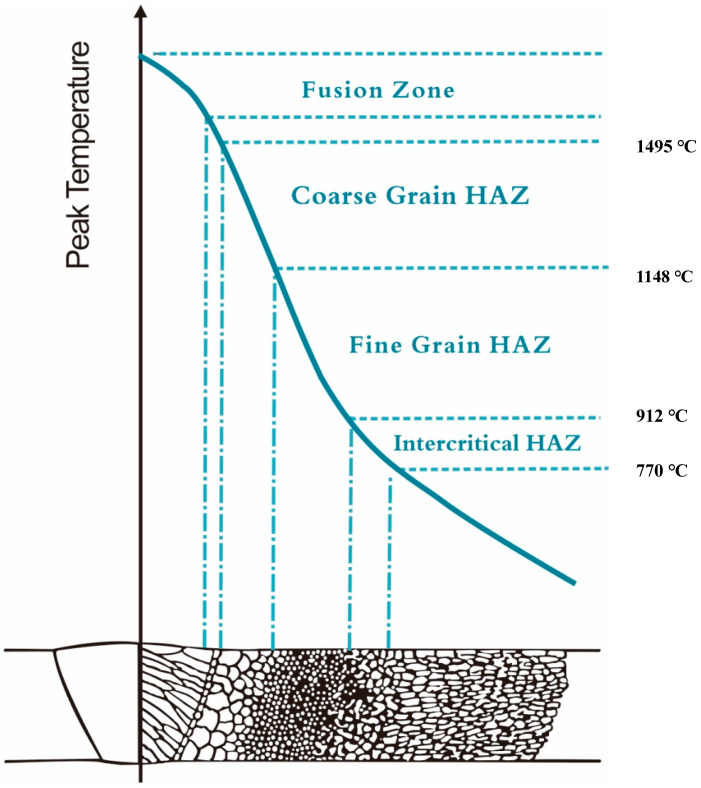
Peak temperature corresponding to different weld regions.

The welding thermal simulation involves controlling the peak temperature and cooling rate to replicate the welding thermal cycle process in different heat-affected zones. Based on the peak temperature profiles of different welding regions in carbon steel as shown in Figure 11 [20], we designed heating and subsequent air-cooling processes to simulate the thermal cycles in different welding locations. We selected five peak temperatures: 850 °C, 950 °C, 1050 °C, 1100 °C, and 1200 °C. The 1200 °C temperature represents the coarse-grain heat-affected zone, while 1100 °C, 1050 °C, and 950 °C represent the fine-grain heat-affected zone, and 850 °C represents the intercritical heat-affected zone.

**Figure 12:**
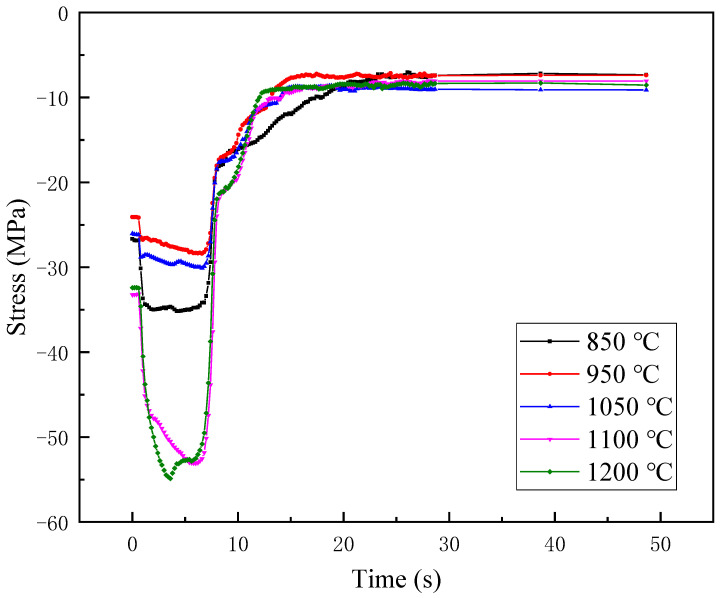
Load variation of base metal of #1 repaired tubing at different simulated peak temperatures.

The thermal simulation test was conducted on the #1 tubing to verify the microstructure of the weld. The two ends of the sample were clamped and were heated to 850 °C, 950 °C, 1050 °C, 1100 °C, and 1200 °C in 10 s intervals, respectively, and then air cooled. The load was recorded during the heating test, and the microstructure and hardness were inspected after the test. Figure 12 shows the load variation when heated to different simulated peak temperatures. The sample is first thermally expanded, and since the two ends are constrained, the sample is subjected to compressive stress, which continues to increase. When air-cooled, the sample shrinks; the sample is subjected to tensile stress; and the load rises in reverse. When heated to 1100 °C and 1200 °C, the maximum compressive stress reaches 55 MPa. After cooling, the maximum stress variation is about 45 MPa.

**Figure 13:**
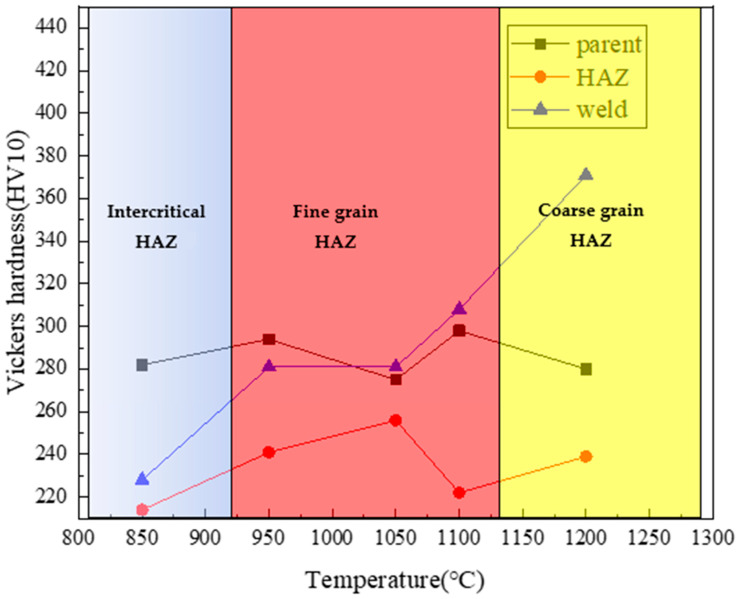
Microhardness value of thermally simulated samples at different peak temperatures.

**Figure 14:**
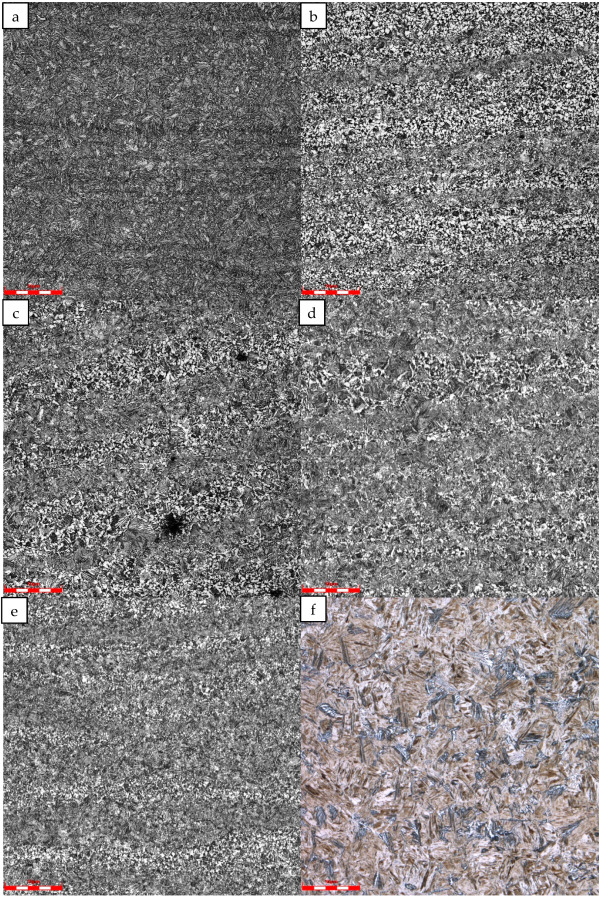
Optical microstructure of #1 repaired tubing base material at different simulated peak temperatures: (**a**) base material, (**b**) 850 °C, (**c**) 950 °C, (**d**) 1050 °C, (**e**) 1100 °C, and (**f**) 1200 °C.

The hardness and microstructure of the tubing base metal at different simulated peak temperatures after thermal simulation are shown in Figure 13 and Figure 14. The results show that when cooling from 850 °C, the tempered sorbite of the parent metal of the tubing transforms into ferrite, pearlite, and bainite, which exhibit a low hardness value, 228HV10. When cooling from 950, 1050, and 1100 °C, the tempered sorbite transforms into bainite, ferrite, and pearlite, exhibiting relative high hardness values, 281~308 HV10. When cooling from 1200 °C, the base metal transforms into martensite with a little granular bainite, exhibiting the highest hardness value of 371HV10.

According to the International Institute of Welding, the maximum Vickers hardness in the heat-affected zone (HAZ) should not exceed 350HV10, and the presence of martensitic structure should be avoided [21,22]. From the thermal simulation results, it is observed that martensitic structure appears in the coarse-grained heat-affected zone (CGHAZ) with a maximum hardness of 371HV10, which is higher than the specified limit of 350HV10. This indicates poor weldability of the material and an increased susceptibility to cold cracking.

### 4.3. Root Cause Analysis

According to the metallographic and hardness results of the fracture section, the microstructure at the weld toe is martensite with a hardness of 476 HV10. Near the weld in the heat-affected zone of the tubing is granular bainite with a hardness of 361 HV10. The microstructure of the base material is tempered sorbite, with a hardness of about 320 HV10. During the welding process, the cooling speed at different positions is different; the microstructure formed is different; and the volume of the microstructure changes differently, so internal stress will be formed. The lath-like martensite structure at the weld toe is a typical hard and brittle structure of carbon steel, which seriously reduces the toughness of the welded joint, leading to cold cracks [23,24] in the heat-affected zone, resulting in failure. To prevent cold cracks, the welding consumables could be dried; the base metal and welding consumables could be degreased and cleaned; and the residual stress could be eliminated by heat treatment after welding.

To sum up, the thread at the crack failure location was not tightened to the specified position; leakage occurred during the pressure test; and welding was used to repair the leakage. However, the P110 oil pipe has a high carbon equivalent, poor weldability, and martensitic structure formed at the weld toe, leading to the occurrence of cold cracks in the heat-affected zone, resulting in failure.

### 4.4. Discussion

Oil tubing and pipelines are manufactured according to different standards, namely API 5CT and API 5L. These two types of pipes have different service environments and requirements.

Oil tubing is primarily used for oil and gas extraction and typically employs threaded connections. Therefore, the API 5CT standard focuses more on the performance and reliability of oil tubing during the extraction process. This standard covers requirements for tubing dimensions, materials, mechanical properties, and chemical composition, ensuring the tubing’s ability to withstand factors such as high pressure, high temperature, and corrosion in harsh oil and gas extraction environments.

Pipelines, on the other hand, are primarily used for oil and gas transportation and commonly utilizes welded connections. As a result, the API 5L standard emphasizes the weldability and reliability of pipelines during the transportation process. This standard covers requirements for pipeline dimensions, materials, mechanical properties, and chemical composition, as well as welding performance, ensuring good weldability and suitability for handling pressures, temperatures, and other mechanical demands in the transportation process.

In some oil fields in China, old and discarded oil tubing is inspected and repaired for use as transmission pipelines. When using oil tubing as transmission pipelines, it is important to consider that during the installation of pipelines, it is difficult to achieve a specified torque on the pipe threads with wrenches. Consequently, ensuring a certain level of sealing becomes challenging. Therefore, it is not recommended to use repaired oil tubing for transporting hydrogen sulfide gas or in high-pressure gas transmission environments.

In the case of pressure testing leaks, considering the higher carbon content and poor weldability of oil tubing, it is advisable to perform welding after preheating and drying, followed by regular inspections to ensure their integrity and to prevent failures.

## 5. Conclusions and Recommendations

In this study, repaired tubing was used as a gas transmission pipeline, and after eight months of operation, it cracked and failed. Macro-inspection, chemical composition analysis, mechanical performance tests, microstructure characterization, fracture morphology, and thermal simulation tests were carried out to find the root cause of the failure, and the following conclusions are obtained:

(1) Macroscopic analysis shows that the thread of the cracked tubing is not tightened to the specified position; the connection between the tubing and the coupling is welded in a circumferential direction; and cracks occur in the heat-affected zone of the weld.
(2) The results of chemical composition and mechanical testing show that the chemical composition, the tensile performance, and the Charpy impact of the tubing meet the requirements of API 5CT for P110 material; no abnormalities are found in the metallographic structure. The microstructure at the weld toe of the fracture is martensite, and the hardness is 476 HV10.
(3) Based on the thermal simulation verification test, when the material of the tubing cools from 1200 °C, which is located in the coarse HAZ temperature zone, the base metal transforms into martensite with a little granular bainite, exhibiting the greatest hardness value of 371 HV10.
(4) The reasons for the cracking and failure of the tubing are that the P110 repaired tubing has a high carbon equivalent and poor weldability. During the welding process, martensitic structure is formed at the weld toe, and cold cracks appear in the heat-affected zone, resulting in failure.

To avoid the reoccurrence of such failure, the following recommendations were proposed:

(1) It is not suitable to use repaired tubing for medium transportation under the conditions of high-pressure gas transmission.
(2) When repaired tubing is used for gathering and transportation construction, special measures should be taken to ensure that the thread is screwed up to the designated position.
(3) To weld the repair tubing, the welding consumables could be dried; the base metal and welding consumables could be degreased and cleaned; and the residual stress could be eliminated by heat treatment after welding.

## Figures and Tables

**Table 1:** Chemical composition of #1, #2 tubing, and coupling (wt.%).

Element	C	Si	Mn	P	S	Cr	Mo	Ni	Nb	V	Ti	Cu
#1 tubing	0.27	0.24	1.44	0.01	0.0046	0.093	0.018	0.022	0.0007	0.0055	0.0024	0.032
#2 tubing	0.26	0.21	0.48	0.0066	0.0011	0.52	0.8	0.03	0.031	0.072	0.0027	0.046
coupling	0.25	0.23	0.96	0.012	0.0065	0.71	0.19	0.02	0.0009	0.0049	0.0026	0.036
API 5CT requirement for P110	/	/	/	≤0.03	≤0.03	/	/	/	/	/	/	/
API 5CT requirement for C110	≤0.35	/	≤1.20	≤0.02	≤0.005	0.4~1.5	0.25~1.0	≤0.99	/	/	/	/

**Table 2:** The tensile test results.

Sample	Tensile Strength /MPa	Yield Strength /Mpa 0.6%EUL	A/%
#1 tubing	958	895	16
	965	907	17
#2 tubing	857	802	25
	861	808	24
API 5CT requirement for P110	≥862	758~965	/
API 5CT requirement for C110	≥793	758~828	/

**Table 3:** The Charpy impact test results.

Sample	Absorption Energy for Longitudinal Sample/J	
	Single Value	Average
#1 tubing	56, 57, 55	56
#2 tubing	81, 82, 81	81
API 5CT requirement for P110	/	≥41
API 5CT requirement for C110	/	≥41

**Table 4:** The Rockwell hardness test results.

Sample	Test Result (HRC)		
#1 tubing	First quadrant	Outside wall location	28.1, 28.6, 27.8
		Mid-wall location	28.8, 30.4, 30.8
		Inside wall location	28.7, 29.8, 30.2
	Second quadrant	Outside wall location	28.3, 28.6, 29.3
		Mid-wall location	29.7, 31.0, 30.8
		Inside wall location	30.1, 29.7, 29.7
	Third quadrant	Outside wall location	27.1, 29.3, 28.2
		Mid-wall location	28.4, 27.5, 28.6
		Inside wall location	28.9, 29.2, 29.9
	Fourth quadrant	Outside wall location	29.1, 27.8, 29.1
		Mid-wall location	27.6, 28.6, 28.8
		Inside wall location	29.1, 29.8, 30.2
#2 tubing	First quadrant	Outside wall location	23.1, 24.3, 25.2
		Mid-wall location	25.0, 23.7, 24.8
		Inside wall location	25.7, 26.3, 26.1
	Second quadrant	Outside wall location	25.0, 25.4, 25.0
		Mid-wall location	23.6, 25.4, 25.0
		Inside wall location	25.0, 23.9, 25.2
	Third quadrant	Outside wall location	22.3, 24.8, 25.0
		Mid-wall location	25.1, 25.3, 26.3
		Inside wall location	24.8, 26.1, 26.0
	Fourth quadrant	Outside wall location	25.6, 25.0, 25.3
		Mid-wall location	24.3, 24.8, 25.2
		Inside wall location	25.6, 26.3, 26.4
API 5CT requirement for C110			<30.0

**Table 5:** Cross-sectional hardness test results at cracks (HV10).

Region	Coupling										
No.	1	2	3	4	5	6	7	8	9	10	11
Hardness	317	313	315	322	327	/	/	/	/	/	/
**Region**	**HAZ near coupling**										
No.	1	2	3	4	5	6	7	8	9	10	11
Hardness	308	328	334	329	279	391	298	316	341	358	/
**Region**	**HAZ near coupling near weld**										
No.	1	2	3	4	5	6	7	8	9	10	11
Hardness	234	221	221	224	249	/	/	/	/	/	/
**Region**	**Weld**										
No.	1	2	3	4	5	6	7	8	9	10	11
Hardness	233	218	403	193	211	197	307	223	214	229	223
**Region**	**#1 tubing**										
No.	1	2	3	4	5	6	7	8	9	10	11
Hardness	329	333	333	328	320	337	329	329	315	327	/
**Region**	**#1 tubing near the fracture**										
No.	1	2	3	4	5	6	7	8	9	10	11
Hardness	317	324	329	328	295	308	321	319	/	/	/
**Region**	**#1 tubing around the crack**										
No.	1	2	3	4	5	6	7	8	9	10	11
Hardness	254	476	361	356	/	/	/	/	/	/	/

## Data Availability

Data available on request due to restrictions eg privacy or ethical.
